# Efficacy of beta-blockers on blood pressure control and morbidity and mortality endpoints in hypertensives of African ancestry: an individual patient data meta-analysis

**DOI:** 10.3389/fcvm.2023.1280953

**Published:** 2024-01-23

**Authors:** Nqoba Tsabedze, R. Darshni Naicker, Sanaa Mrabeti

**Affiliations:** ^1^ Division of Cardiology, Department of Internal Medicine, Faculty of Health Sciences, University of the Witwatersrand, Johannesburg, South Africa; ^2^ Medical Department, Healthcare Division, Merck Pty Ltd, Modderfontein, South Africa; ^3^ Medical Affairs EMEA, Merck Serono Middle East FZ-LLC, Dubai, United Arab Emirates

**Keywords:** hypertension, beta-blocker, antihypertensive, individual patient data meta-analysis, cardiovascular outcomes, Africa, blood pressure (BP)

## Abstract

**Introduction:**

Compared with first-line antihypertensives, beta-blockers (BB) have been reported to lower the central aortic blood pressure suboptimally and are associated with increased stroke risk. This observation has not been investigated in hypertensives of African ancestry. We hypothesised that an individual patient data meta-analysis (IPD-MA) on the efficacy of second- or third-generation beta-blockers (STGBBs) in hypertensives of African descent may provide new insights.

**Methods:**

A single-stage IPD-MA analysed the efficacy of STGBB in lowering the mean arterial blood pressure and reducing the composite outcomes: cardiovascular death, stroke, and myocardial infarction.

**Results:**

A total of 11,860 participants from four randomised control trials were included in the analysis. Second- or third-generation beta-blockers reduced the mean arterial pressure by 1.75 mmHg [95% confidence interval (CI):1.16–2.33; *P* < 0.001] in all participants included in the analysis, and by 1.93 mmHg (95% CI: 0.86–3.00; *P* < 0.001) in hypertensive Africans. In patients with established cardiovascular disease, where the benefits of BB therapy are well established, STGBBs were associated with an adjusted odds ratio of 1.33 (95% CI: 1.06–1.65; *P* = 0.015) of the composite outcome, most likely due to confounding. Similarly, the risk of total myocardial infarction was 1.76 times higher (95% CI: 1.15–2.68; *P* = 0.008) in hypertensives of African ancestry on STGBBs.

**Conclusion:**

The STGBBs reduced the mean arterial pressure comparably to other antihypertensives, and they were not associated with an increased risk of stroke.

## 1 Introduction

Beta-blockers are classified by generation as follows: first-generation beta-blockers refer to non-selective beta-blockers without any additional vasodilatory effects; second-generation beta-blockers generally refer to β_1_-selective beta-blockers; and third-generation beta-blockers refer to beta-blockers that possess additional vasodilatory action, often mediated by the release of nitric oxide.

Beta-blockers (BBs) are a pharmacologically heterogeneous class of drugs widely used over the past five decades (1). The ability of BBs to modulate the sympathetic nervous system (SNS) through the adrenergic blockade of β_1_ and β_2_ receptors, and variable vasodilatory properties, position these agents as efficacious first-line antihypertensives. However, several clinical guidelines for the management of hypertension have recently withdrawn BBs as a first-line therapy (2–5), citing that, compared with other classes of antihypertensives, BBs reduce central aortic pressure suboptimally and offer less protection against fatal and non-fatal strokes (6).

This contentious decision to withdraw BB as a first-line therapy has been found to be controversial and met with some opposition. No prospective randomised control trials (RCTs) have investigated the efficacy of, specifically, second or third-generation beta-blockers (STGBBs) in reducing morbidity and mortality in hypertensives. Furthermore, individuals of African ancestry are usually underrepresented in RCTs (7). Yet, they have a higher prevalence of hypertension and are more likely to experience target organ damage caused by poorly controlled hypertension (8–11). This increase in hypertension-related morbidity and mortality may be because of the interplay between biological factors and myriad social drivers (12–15). We conducted an individual patient data meta-analysis (IPD-MA) of RCTs evaluating the efficacy of STGBBs in hypertensives of African ancestry in lowering blood pressure and the risk of cardiovascular death, fatal and non-fatal myocardial infarction (MI), and strokes.

## 2 Materials and methods

The study protocol was registered on the International Prospective Register for systematic reviews (PROSPERO ID = CRD42022344733). This IPD-MA adhered to the Preferred Reporting Items for Systematic Reviews and Meta-Analyses (PRISMA) for individual patient data systematic reviews (PRISMA-IPD) statement (16).

### 2.1 Selection of randomised control trials and participants included in the individual patient data meta-analysis

Following a prespecified search strategy, we performed a comprehensive systematic literature search in multiple clinical trial registries, search engines, and dataset repositories. (Supplementary material 1). All published and unpublished RCTs that assessed the efficacy of BBs, or antihypertensives, including STGBBs, compared with placebo, standard-of-care (SoC), or other antihypertensives (including first-generation BBs) in hypertensive participants were eligible for inclusion. Eligible RCTs were required to report the efficacy of BBs in lowering blood pressure and outcomes such as cardiovascular death, MI, and strokes in hypertensive individuals. Furthermore, RCTs required a proportion of the study participants to be of African descent. We excluded RCTs with a follow-up duration shorter than 1 year, participants younger than 18 years of age, studies conducted on healthy volunteers, and those without participants of African descent.

The risk of confounding was minimised by excluding participants with a history of previous major cardiovascular events. Participants were excluded from the analysis if they had a prior history of MI, congestive heart failure (CHF), arrhythmia, history of coronary revascularisation, and transient ischaemic attacks (TIAs) or stroke. Two reviewers independently examined the eligibility of all potential RCTs and assessed the risk of bias (RoB). Differences were resolved through discussion and consensus, and, where required, a third clinical expert arbitrator was consulted.

### 2.2 Individual patient data collection and assessment

The individuals listed as corresponding authors were contacted via email for each RCT study that met the inclusion criteria (Supplementary material 2). The integrity of the obtained datasets was checked for consistency and completeness (17). Also, summary statistics of received datasets were matched with published results, and internal summary statistics were generated as part of the IPD-MA to identify discrepancies. The variables of interest were extracted, standardised, and merged into a single dataset, ensuring that standard scales and definitions were used (Supplementary material 3). The study participants self-identified their ethnicity. The RoB 2 tool adapted for IPD-MA was used to assess the RoB in each study included in the meta-analysis (18, 19) (Supplementary material 4). All datasets included in this study were obtained from repositories within the National Institute of Health umbrella or Vivli.org. No IPD integrity or RoB 2 concerns were identified (Supplementary material 4). The internal and external summary statistics of the individual datasets matched, verifying the accuracy of the datasets employed in the final analysis.

### 2.3 Outcomes and treatment group allocation

The efficacy of BBs was assessed by evaluating the rate of cardiovascular mortality, myocardial infarction, and non-fatal and fatal strokes in hypertensives included in the IPD-MA prescribed STGBB compared with those on a placebo or first-generation BB therapy. To estimate the risk of composite events, odds ratios (OR) were extracted from each study, and the reduction in blood pressure (BP) was calculated by measuring the difference between the baseline and exit mean arterial blood pressure (MAP).

The treatment arm consisted of the whole BB class, which was partitioned into a newer second- and third-generation BB (STGBB group), defined by the β_1_ adrenergic receptor affinity (second generation) and/or vasodilatory properties mediated by the release of Nitric oxide (third generation) (20), and their respective individual components of non-selective, selective β-receptor properties and vasodilatory properties. For studies that did not have a BB treatment arm, we reviewed the documented concomitant medication to allocate participants to their respective treatment groups. Participants were included in the STGBB group if they were on a BB for at least 18 months.

### 2.4 Statistical analysis

The respective RCT data were combined, creating a standardised IPD dataset. Exploratory and descriptive analysis preceded the one-stage IPD-MA. Categorical variables were summarised with frequencies and percentages. The mean and standard deviation and the median with minimum and maximum values were used to summarise numerical variables. The intraclass coefficient (ICC) was calculated by quantifying the degree to which participants within RCTs were alike, based on proportional variance. The generalised linear mixed effects model (GLMM) building process, evaluating both the fit of random intercept and/or random slopes in the modelling process was used. Known confounders (age, gender, ethnicity, diabetes, and smoking) for cardiovascular death were included in the analysis if they were statistically significant, clinically relevant, or the inclusion improved the model's goodness of fit (GoF) statistics. The iterative process assessed each covariate in this manner. Assumptions of GLMMs were robustly evaluated to curb any violations that may negatively influence the validity of the results (Supplementary material 5). Univariate logistic regression analysis was conducted and the odds ratios were adjusted for confounding by including covariates traditionally regarded as predictors of cardiovascular outcomes. Confidence intervals (CIs) were set at 95% and a *P*-value <0.05 was set as a threshold for statistical significance. Sensitivity analysis quantifying study heterogeneity and trends were performed as part of the statistical analysis. All data manipulation and analyses were done in R (version 4.2.1) (21) using the *Tidyverse* (22) and *lme4* (23) packages.

### 2.5 Role of the funding

The funder of this research (Merck) had no role in data collection, statistical analysis, data wrangling, data interpretation, and manuscript writing. The funders gave their consent towards the publication of the manuscript.

### 2.6 Ethics

Permission to conduct the study was obtained from the University of the Witwatersrand Human Research Ethics Committee (Medical), ethics clearance certificate number: W-CBP-211102-01.

## 3 Results

### 3.1 Study cohort selection and baseline characteristics

**Figure 1:**
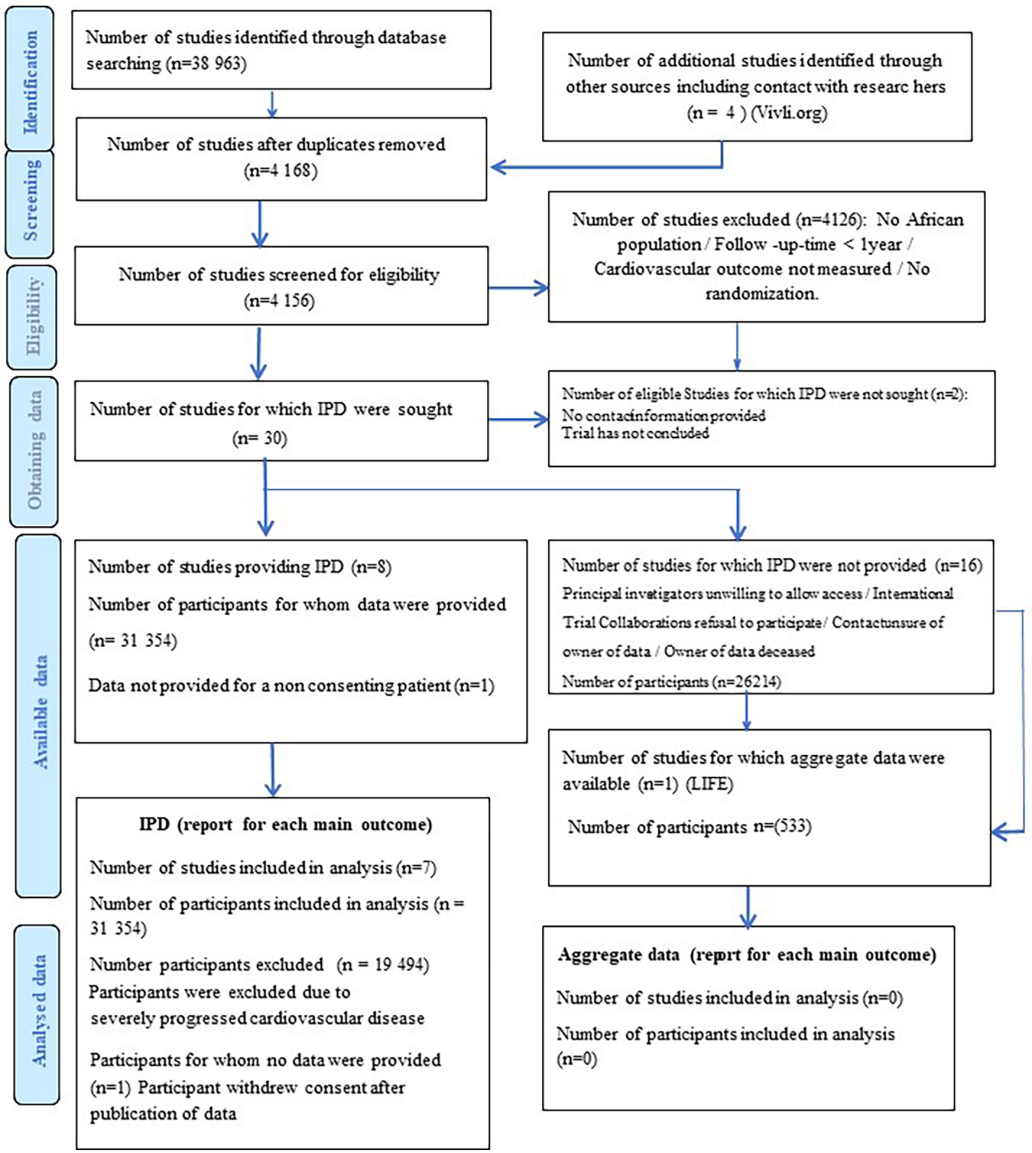
PRISMA flow chart showing the selection of studies and participants included in the meta-analysis.

The search strategy resulted in 30 eligible RCTs (Figure 1). All authors listed in the respective trials were contacted, of which 23 authors responded. Of the 30 RCT datasets requested, access was granted to seven datasets. We excluded 19,494 participants with established severe CVD. The final study cohort in the IPD-MA comprised 11,860 hypertensives from four RCTs (Table 1). The STGBB arm had 3,864 (32.6%) hypertensives with a mean age of 66.4 ± 9.2 years, of which 1,339 (34.7%) hypertensives were of African ancestry. Similarly, the non-STGBB arm had 2,541 (31.8%) hypertensives of African ancestry. The STGBB arm had more individuals who had been prescribed concomitant antihypertensive medication; with diuretics prescribed to 3,131 (81.1%) vs. 3,781 (47.3%) in the non-STGBB arm. Of note, 2,075 (53.7%) participants in the STGBB arm were prescribed calcium channel blockers (CCB), compared with the 2,827 (35.4%) in the non-STGBB arm. The baseline mean systolic and diastolic blood pressures were comparable in both arms of the study. Table 2 depicts the type of BBs prescribed in each RCT.

**Table 1:** Standardised demographic and clinical characteristics of patients included in the meta-analysis stratified by the trial providing the patient data.

Study name	Systolic hypertension in the elderly program (24)		Action to control cardiovascular risk in diabetes (25)		Systolic blood pressure intervention trial (26)		African-American study of kidney disease and hypertension (27)		Total	
Acronym	SHEP (*n* = 1,497)		ACCORD (*n* = 3,053)		SPRINT (*n* = 6,802)		AASK (*n* = 508)		*N* = 11,860	
	STGBB	No STGBB	STGBB	No STGBB	STGBB	No STGBB	STGBB	No STGBB	STGBB	No STGBB
Number randomised	389	1,108	660	2,393	2,609	4,193	206	302	3,864	7,996
Age										
Mean (SD)	71.4 (6.37)	71.8 (6.42)	63.3 (5.854)	62.8 (5.89)	67.3 (2.93	66.7 (9.29)	55.1 [10.3]	54.6 (10.7)	66.4 (9.20)	65.8 (8.86)
Median (min, max)	71.0 (60.0, 90.0)	71.0 (60.0, 91.0)	62.3 (55.0, 78.5)	61.9 (55.0, 79.3)	66.0 (50.0, 90.0)	65.0 (46.0, 90.0)	56.5 (24.0, 70.0)	56.0 (21.0, 70.0)	65.5 (24.0, 90.0)	65.0 (21.0, 91.30)
Gender										
Male	179 (46.0%)	488 (44.0%)	333 (50.5%)	1,092 (45.6%)	1,512 (58.0%)	2,675 (63.8%)	117 (56.8%)	166 (55.0%)	2,141 (55.4%)	4,421 (55.3%)
Female	210 (54.0%)	620 (54.0%)	327 (49.5%)	1,301 (54.4%)	1,097 (42.0%)	1,518 (36.2%)	89 (43.2%)	136 (45.0%)	1,723 (44.6%)	3,575 (44.7%)
Race										
White	298 (76.6%)	871 (78.6%)	379 (57.4%)	1,357 (56.7%)	1,607 (61.6%)	2,580 (61.5%)	0 (0%)	0 (0%)	2,284 (59.1%)	4,808 (60.2%)
African	56 (14.4%)	162 (14.6%)	169 (25.6%)	612 (25.6%)	908 (34.9%)	1,465 (34.9%)	206 (100.0%)	302 (100.0%)	1,339 (34.7%)	2,541 (31.8%)
Other	35 (9.0%)	75 (6.8%)	112 (17.0%)	424 (17.7%)	94 (3.6%)	148 (3.5%)	0 (0%)	0 (0%)	241 (6.3%)	647 (8.1%)
Average follow-up time (years)	4.58 (0.798)	4.53 (0.800)	5.06 (1.18)	4.68 (1.48)	3.95 (0.703)	3.71 (1.03)	4.51 (1.23)	4.51 (1.23)	4.24 (0.950)	4.15 (1.25)
History of diabetes										
No	339 (87.1%)	1,017 (91.8%)	0 (0%)	0 (0%)	2,565 (98.7%)	4,144 (98.6%)	206 (100%)	302 (100%)	3,120 (80.7%)	5,453 (68.2%)
Yes	50 (12.9%)	91 (8.2%)	660 (100%)	2,393 (100%)	34 (1.3%)	59 (1.4%)	0 (0%)	0 (0%)	744 (19.3%)	2,543 (31.8%)
Current smoking										
No	355 (91.3%)	966 (87.2%)	77 (11.7%)	298 (12.5%)	2,253 (86.7%)	3,623 (86.2%)	155 (75.2%)	235 (77.8%)	2,850 (73.8%)	5,122 (63.9%)
Yes	34 (8.7%)	142 (12.8%)	583 (88.3%)	2,095 (87.5%)	346 (13.3%)	580 (13.8%)	51 (24.8%)	51 (24.8%)	1,014 (26.2%)	2,884 (36.2%)
Concomitant antihypertensive medication										
Diuretic use										
No	0 (0%)	1,108 (100%)	94 (14.2%)	1,529 (63.9%)	433 (16.7%)	1,276 (30.4%)	206 (100%)	302 (100%)	733 (19.0%)	4,215 (52.6%)
Yes	389 (100%)	0 (0%)	566 (85.8%)	864 (36.1%)	2,176 (83.4%)	2,917 (69.6%)	0 (0%)	0 (0%)	3,131 (81.0%)	3,781 (47.3%)
ACE-inhibitor use										
No	389 (100%)	1,108 (100%)	285 (43.2%)	1,502 (62.8%)	1,297 (49.7%)	2,064 (49.2%)	206 (100%)	95 (31.5%)	2,177 (56.4%)	4,769 (59.6%)
Yes	0 (0%)	0 (0%)	375 (56.6%)	891 (37.2%)	1,312 (50.3%)	2,129 (50.8%)	0 (0%)	207 (68.5%)	1,687 (43.7%)	3,227 (40.4%)
Calcium channel blocker use										
No	389 (100%)	1,108 (100%)	405 (61.4%)	2,065 (86.3%)	789 (30.2%)	1,789 (42.7%)	206 (100%)	207 (68.5%)	1,789 (46.3%)	5,169 (64.6%)
Yes	0 (0%)	0 (0%)	255 (38.6%)	328 (13.7%)	1,820 (69.8%)	2,404 (57.3%)	0 (0%)	95 (31.5%)	2,075 (53.7%)	2,827 (35.4%)
Angiotensin II receptor blocker										
No	389 (100%)	1,108 (100%)	396 (60.0%)	1,968 (86.3%)	1,470 (56.3%)	2,673 (63.8%)	206 (100%)	302 (100%)	2,461 (63.7%)	6,051 (75.7%)
Yes	0 (0%)	0 (0%)	264 (40.0%)	425 (13.7%)	1,139 (43.7%)	1,520 (36.2%)	0 (0%)	0 (0%)	1,401 (36.3%)	1,945 (24.3%)
Blood pressure (mmHg)										
Baseline systolic										
Mean (SD)	173 (13.2)	171 (13.4)	143 (16.2)	139 (15.1)	141 (16.3)	139 (15.0)	146 (21.7)	146 (20.8)	145 (18.8)	144 (18.8)
Median (min, max)	171 (134, 217)	171 (130, 217)	143 (98.0, 228)	137 (96.0, 202)	140 (87.0, 231)	138 (93.0, 214)	145 (99.0, 206)	143 (98.0, 228)	142 (87.0, 231)	141 (93.0, 228)
Baseline diastolic										
Mean (SD)	76.5 (9.75)	80.7 (10.0)	94.3 (13.1)	76.5 (9.75)	79.2 (12.3)	79.3 (11.4)	93.6 (12.7)	94.3 (13.1)	80.0 (12.2)	79.2 (11.2)
Median (min, max)	76.0 (20.00, 99.0)	81.0 (0, 99.0)	96.0 (44.0, 146)	76 (45.0, 113)	79.0 (42.0, 134)	79.0 (42.0, 126)	96.0 (62.0, 134)	96.0 (44.0, 146)	80 (42.0, 134)	79.0 (20.0, 146)

The studies are stratified by the use of STGBBs. Hypertension is often treated with combination/multiple agents. Both the STGBB and non-STGBB groups could be on multiple antihypertensives. The only difference is the fact that the STGBB group were on STGBB.

**Table 2:** Beta-blocker prescription in each randomised control trial.

	Randomised control trial			
	AASK (*n* = 206)	ACCORD (*n* = 681)	SHEP (*n* = 389)	SPRINT (*n* = 2,878)
Propranolol (first-generation BB)				29 (1.0%)
Nadolol (first-generation BB)				3 (0.1%)
Metoprolol (first-generation BB)	206 (100%)	556 (81.6%)		1 446 (50.2%)
Acebutolol (second-generation BB)				0 (0%)
Atenolol (second-generation BB)			389 (100%)	1 188 (41.3%)
Bisoprolol (second-generation BB)				13 (0.5%)
Carvedilol (third-generation BB)		105 (15.4%)		153 (5.3%)
Labetalol (third-generation BB)				43 (1.5%)
Nebivolol (third-generation BB)				3 (0.1%)
Other beta-blockers		20 (3.0%)		

This table reports on the BB used per trial and not what was finally analysed in the IPD-MA.

### 3.2 Study outcomes

Hypertensives included in the IPD-MA had a mean follow-up duration of 4.13 years. The STGBB arm recorded 217 (5.6%) primary events compared with 374 (4.7%) events in the non-STGBB arm (Table 3). Myocardial infarction occurred in 134 (3.5%) and 178 (2.2%) hypertensives in the STGBB and non-STGBB arms, respectively. In the included RCTs, strokes were more likely to be non-fatal, occurring in 81 (2.1%) hypertensives in the STGBB arm, compared with 140 (1.7%) in the non-STGBB arm.

**Table 3:** Primary and secondary outcomes stratified by randomised control trial.

Study name	Systolic hypertension in the elderly program		Action to control cardiovascular risk in diabetes		Systolic blood pressure intervention trial		African-American study of kidney disease and hypertension		Total	
Acronym	SHEP (*n* = 1,497)		ACCORD (*n* = 3 053)		SPRINT (*n* = 6,802)		AASK (n = 508)			
	STGBB	No STGBB	STGBB	No STGBB	STGBB	No STGBB	STGBB	No STGBB	STGBB	No STGBB
Total cardiovascular mortality	4 (1.0%)	11 (1.0%)	1 (0.2%)	46 (1.9%)	9 (0.3%)	23 (0.5%)	5 (2.4%)	3 (1.0%)	19 (0.5%)	83 (1.0%)
Total stroke	14 (3.6%)	53 (4.8%)	14 (2.1%)	40 (1.7%)	51 (2.0%)	51 (1.2%)	8 (3.9%)	10 (3.3%)	87 (2.3%)	154 (1.9%)
Total myocardial infarction	5 (1.3%)	22 (2.0%)	29 (4.4%)	88 (3.7%)	96 (3.7%)	62 (1.5%)	4 (1.9%)	8 (2.6%)	134 (3.5%)	178 (2.2%)
Primary outcome										
No	369 (94.9%)	1,028 (92.8%)	617 (93.5%)	2,234 (93.4%)	2,460 (94.7%)	4,087 (97.2%)	191 (92.7%)	283 (93.7%)	3,637 (94.4%)	7,632 (95.3%)
Yes	20 (5.1%)^a^	80 (7.2%)	43 (6.5%)	159 (6.6%)	139 (5.3%)	116 (2.8%)	15 (7.3%)	19 (6.3%)	217 (5.6%)	374 (4.7%)
Total cardiovascular events										
No	348 (89.5%)	935 (84.4%)	617 (93.5%)	2,234 (93.4%)	2,408 (92.3%)	4,053 (96.7%)	190 (92.2%)	281 (93.0%)	3,563 (92.2%)	7,503 (93.8%)
Yes	41 (10.5%)	173 (15.6%)	43 (6.5%)	159 (6.6%)	201 (7.7%)	140 (3.3%)	16 (7.6%)	21 (7.0%)	301 (7.8%)	493 (6.2%)
Stroke										
Non-fatal	13 (3.3%)	52 (4.7%)	14 (2.1%)	34 (1.4%)	48 (1.8%)	44 (1.0%)	6 (2.9%)	10 (3.3%)	81 (2.1%)	140 (1.7%)
Fatal	2 (0.5%)	1 (0.1%)	0 (0.0%)	9 (0.4%)	3 (0.1%)	7 (0.2%)	2 (1.0%)	0 (0%)	7 (0.2%)	17 (0.2%)
Total	14 (3.6%)^a^	53 (4.8%)	14 (2.1%)	40 (1.7%)	51 (2.0%)	51 (1.2%)	8 (3.9%)	10 (3.3%)	87 (2.3%)	154 (1.9%)
Myocardial infarction										
Non-fatal	5 (1.3%)	18 (1.6%)	29 (4.4%)	88 (3.7%)	93 (3.6%)	50 (1.2%)	4 (1.9%)	7 (2.3%)	131 (3.4%)	163 (2.0%)
Fatal	0 (0%)	4 (0.4%)	0 (0%)	4 (0.2%)	3 (0.1%)	12 (0.3%)	0 (0%)	1 (0.3%)	3 (0.1%)	21 (0.3%)
Total	5 (1.3%)	22 (2.0%)	29 (4.4%)	88 (3.7%)	96 (3.7%)	62 (1.5%)	4 (1.9%)	8 (2.6%)	134 (3.5%)	178 (2.2%)
Congestive heart failure										
Non-fatal	7 (1.8%)	33 (3.0%)	14 (2.1%)	39 (1.6%)	79 (3.0%)	41 (1.0%)	6 (2.9%)	3 (1.0%)	106 (2.7%)	116 (1.5%)
Fatal	1 (0.3%)	2 (0.2%)	0 (0%)	13 (0.5%)	3 (0.1%)	4 (0.1%)	0 (0%)	0 (0%)	4 (0.1%)	19 (0.2%)
Total	8 (2.1%)	34 (3.1%)^a^	14 (2.1%)	52 (2.2%)	82 (3.1%)	45 (1.1%)	6 (2.9%)	3 (1.0%)	110 (2.8%)	134 (1.5%)
Blood pressure (mmHg)										
Exit systolic										
Mean (SD)	147 (18.7%)	155 (18.2)	124 (16.2)	129 (16.1)	131 (16.3)	131 (15.1)	136 (13.0)	135 (13.6)	131 (17.5)	134 (18.3)
Median (min, max)	143 (100, 214)	154 (87.0, 218)	121 (86.0, 206)	128 (79.0, 217)	130 (74.0, 189)	130 (77, 206)	135 (102, 168)	133 (93.3, 177)	130 (74.0, 214)	130 (77.0, 214)
Exit diastolic										
Mean (SD)	81.2 (8.63)	81.0 (8.98)	78.0 (10.5)	76.7 (9.72)	79.3 (12.2)	79.3 (11.1)	93.7 (12.7)	94.3 (13.1)	80.1 (12.1)	79.5 (11.1)
Median (min, max)	82.0 (57.0, 99.0)	81.0 (20.0, 99.0)	78.0 (50.0, 108)	76.0 (45.0, 113)	79.0 (43.0, 123)	79.0 (43.0, 123)	96.0 (62.0, 134)	96.0 (44.0, 146)	80.0 (42.0, 134)	79.0 (20.0, 146)

^a^ The events are not mutually exclusive. For example, participants may have a non-fatal major cardiovascular event followed by a fatal cardiovascular event resulting in the respective sum of events not equalling the totals displayed. The studies are stratified by the use of STGBB. Hypertension is often treated with combination/multiple agents. Both the STGBB and non-STGBB groups could be on multiple antihypertensives. The defining difference is that the STGBB group were on STGBB.

Hypertensives on STGBB were 1.2 times more likely to experience composite primary outcomes (95% CI: 1.02–1.44; *P*-value = 0.028) compared with those in the non-STGBB arm (Table 4). Myocardial infarctions were 1.8 times (95% CI: 1.15–2.68; *P*-value = 0.008) more likely to occur in hypertensives of African ancestry compared to the entire population (95% CI: 1.24–1.96; *P*-value < 0.001) on STGBB. After adjusting the odds ratios for confounders such as age, gender, and smoking (Table 5), there were no statistically significant differences in the risk of composite primary outcomes in the STGBB arm when comparing hypertensives of African ancestry and other racial groups.

**Table 4:** Univariate analysis looking at the STGBB arm compared with the non-use of STGBB.

Outcome	Whole population				African population			
		95% confidence interval				95% confidence interval		
	Odds ratio	Lower	Upper	*P*-value	Odds ratio	Lower	Upper	*P*-value
Cardiovascular mortality								
STGBB	0.47	0.28	0.76	0.003	0.69	0.29	1.49	0.370
Total stroke								
STGBB	1.17	0.9	1.53	0.239	1.13	0.72	1.74	0.595
Total myocardial infarction								
STGBB	1.56	1.24	1.96	<0.001	1.76	1.15	2.68	0.008
Primary outcome								
STGBB	1.22	1.02	1.44	0.028	1.26	0.93	1.7	0.136
Non-fatal stroke								
STGBB	1.20	0.91	1.58	0.193	1.17	0.73	1.84	0.513
Non-fatal myocardial infarction								
STGBB	1.69	1.33	2.13	<0.001	1.80	1.17	2.75	0.007
Total cardiovascular disease								
STGBB	1.29	1.11	1.49	<0.001	1.28	0.98	1.66	0.073
Change in blood pressure (exit-baseline)	Change in blood pressure (mmHg)				Change in blood pressure (mmHg)			
Systolic (mmHg) (ICC = 34%)								
STGBB	−2.4	−3.25	−1.54	<0.001	−3.05	−4.61	−1.49	<0.001
Diastolic (mmHg) (ICC = 41%)								
STGBB	−1.33	−1.83	−0.84	<0.001	−1.71	−2.61	−0.81	<0.001

**Table 5:** Multivariable logistic regression model predicting the primary outcome: total cardiovascular mortality, total stroke, and total myocardial infarction in 11,860 patients included in the meta-analysis.

		95% confidence interval		
	aOR	Lower	Upper	*P*-value
STGBB	1.33	1.06	1.65	0.015
Race				
White	1			
African	1.09	0.85	1.39	0.497
Other	0.68	0.44	1.05	0.080
Age	1.05	1.04	1.06	<0.001
Gender				
Male	1			
Female	0.80	0.67	0.95	0.010
Current smoker				
No	1			
Yes	1.47	1.14	1.89	0.003
STGBB × African race	1.00	0.69	1.46	0.989
STGBB × Other race	1.12	0.53	2.36	0.765

History of diabetes was neither statistically significant nor did it improve the model fit statistics [likelihood ratio test (LRT)], and therefore it was omitted from this model. The adjusted odds ratio (aOR) was controlled for age, gender, and current smoker status.

*N* = 11,860 participants included in this analysis.

### 3.3 Efficacy of second- and third-generation beta-blockers in lowering the mean arterial pressure

Second- and third-generation BBs reduced the MAP in the whole population by 1.75 mmHg (95% CI: 1.16–2.33; *P* < 0.001) compared with 1.93 mmHg (95% CI: 0.86–3.00; *P* < 0.001) in hypertensives of African ancestry (Table 6). The multivariable generalised mixed effects model also demonstrated a statistically significant reduction in the MAP in hypertensives prescribed angiotensin-converting enzyme inhibitors (ACE-I), angiotensin II receptor blockers (ARB), calcium channel blockers, and diuretics. The highest reduction in the MAP was 3.88 mmHg (95% CI: 3.23–4.54; *P* < 0.001) and was associated with diuretic use. In hypertensives of African ancestry, diuretics decreased the MAP by 4.17 mmHg (95% CI: 2.78–5.50; *P* < 0.001). Despite a relatively smaller sample size in the African population, diuretics, ARBs, and STGBBs reduced the MAP more effectively in the African population than in the whole study population.

**Table 6:** Multivariable generalised mixed effects model with the respective RCT as a random effect (random intercept).

	Whole population (*n* = 10,210)				African population (*n* = 3,276)			
		95% confidence interval				95% confidence interval		
	mmHg	Lower	Upper	*P*-value	mmHg	Lower	Upper	*P*-value
(Intercept)	−6.70	−11.80	−1.61	0.01	−6.52	−12.56	−0.48	0.034
STGBB	−1.75	−2.33	−1.16	<0.001	−1.93	−3.00	−0.86	<0.001
ACE-I	−1.67	−2.33	−1.05	<0.001	−1.57	−2.67	−0.48	0.005
Angiotensin II receptor blockers	−2.22	−2.89	−1.54	<0.001	−3.17	−4.44	−1.90	<0.001
Diuretic use	−3.88	−4.54	−3.23	<0.001	−4.17	−5.5	−2.78	<0.001
Calcium channel blockers	−2.61	−3.25	−1.97	<0.001	−1.86	−3.07	−0.66	<0.001

## 4 Discussion

In this IPD-MA, we evaluated the efficacy of STGBBs in reducing the risk of cardiovascular death, strokes, and MI in hypertensives of African ancestry compared with other racial groups on non-STGBBs. The composite primary outcome was higher in the STGBB arm (5.6%), compared with the 4.7% in the non-STGBB arm. In the entire hypertensive study population, including hypertensives of African ancestry, STGBBs were efficacious in reducing the risk of cardiovascular death. Although the trend was evident in African hypertensive, the estimate was not statistically significant. Furthermore, STGBBs had a greater MAP reduction in the African population.

Data supporting the recommendation to withdraw BBs as first-line therapy suggested that BBs failed to reduce the central aortic pressure and were associated with a greater risk of cardiovascular death or stroke (28–31). In this IPD-MA, we found that STGBBs reduced the MAP as efficiently as other antihypertensives in the African population and other racial groups included in the analysis. The findings from our meta-analysis indicate a need for prospective outcomes-driven RCTs to definitively examine the role of STGBBs in treating uncomplicated hypertension.

Beta-blockers prevent complications in patients with hypertension by lowering blood pressure and reducing cardiovascular events with an efficacy similar to other antihypertensives (30, 32–40). In this IPD-MA, we found that STGBBs significantly reduced the MAP in participants of African ancestry. However, the risk of MI was higher in hypertensives of African ancestry who were prescribed STGBB. A meta-analysis by Lindholm et al. published in 2005 evaluating whether BBs should remain first-line agents in the treatment of hypertension found that the relative risk of stroke was 26% higher in participants prescribed atenolol vs. other antihypertensive treatment (41). However, in this study, atenolol was not associated with an increased risk of stroke.

Hypertension is common in individuals of African ancestry and tends to follow a severe course associated with a higher rate of morbidity and mortality (14). The higher prevalence of hypertension in the African population compared with their counterparts has been attributed to biological factors, increased psychological stress, poor socioeconomic status, disparities in salt retention, and a higher rate of obesity among individuals of African ancestry (42, 43). Recent evidence suggests that there may be no racial disparities in the response to antihypertensive treatment (44).

Some of the trials that demonstrated higher adverse events in hypertensives treated with BBs include the Cardiovascular Morbidity and Mortality in the Losartan Intervention For Endpoint (LIFE) Reduction in Hypertension Trial and the Anglo-Scandinavian Cardiac Outcomes Trial—Blood Pressure Lowering Arm (ASCOT-BPLA). The LIFE trial randomised 9,222 hypertensives to atenolol or losartan. After a follow-up duration of 4.8 years, the rate of cardiovascular mortality was 10.6 per 1,000 patient-years of follow-up in the atenolol arm vs. 9.2 per 1,000 patient-years in the losartan arm (28). The stroke rate was also higher in the atenolol arm (14.5 vs. 10.8 per 1,000 patient-years of follow-up). In the ASCOT-BPLA, participants were randomly assigned to either atenolol plus a thiazide diuretic or amlodipine and perindopril. Strokes, cardiovascular events, and all-cause mortality rates were higher in participants randomised to atenolol and a thiazide diuretic (29). Although these studies highlight a higher risk of stroke in patients prescribed BBs, both RCTs had suboptimal dosing of atenolol.

In this IPD-MA, confounding was partially controlled for by excluding participants with previous cardiovascular events and adjusting the odds ratio in the multivariable regression model. For example, we relied on documented baseline clinical history and examination findings to identify high-risk hypertensives with previous MI, strokes, or CHF, requiring exclusion from the study. As such, the significant increase in the risk of MI in hypertensives could be accounted for by including undocumented high-risk hypertensives in the IPD-MA. Although reasonable attempts were made to minimise confounding, the STGBB group was still a higher risk group than the non-STGBB group. The patients in the STGBB group were on more antihypertensive medication than those in the non-STGBB group. This may explain the increased risk of cardiovascular outcomes associated with the STGBB group found in this study.

Individual patient data meta-analysis is considered the most reliable and robust method for obtaining evidence (45). Despite this, most authors prefer conducting systematic reviews and meta-analyses based on aggregated data from various research studies instead of requesting individual participant data from the data custodians. The major drawback of conducting IPD-MA is the unpredictable access to data. Despite receiving a response from 77% of the authors contacted, we could only access data from seven RCTs and eventually only analysed four. Some reasons cited in the literature restricting access to data include a lack of response from the authors or data custodians contacted, operational constraints, staff relocation, and lack of communication (46). Data-sharing policies vary with each country, and authors requesting access to data may be expected to apply for ethical clearance in multiple institutions prior to accessing data. The median time from the first request to accessing data to fully receiving the data can also be excessively long and may take up to 242 days (46). Realising that obtaining data for IPD-MA comes with many challenges, Ventresca et al. recommend requesting data through personal contact, offering incentives such as coauthorship and setting up a data-sharing agreement (47). Furthermore, consenting authors could also deposit de-identified data in a common data repository site. Data confidentiality breach and leakage are some of the key areas that need to be addressed before implementing such data repositories.

## 5 Limitations

This IPD-MA's chief limitation is the incomplete acquisition of IPD from previously conducted RCTs, leading to a smaller sample size. Although generalisability is improved, severe confounding was introduced through the surrogacy BB treatment, since BBs are traditionally prescribed in individuals with established or advanced cardiovascular risk factors. A higher proportion of ACE-I, CCB, ARB, and diuretics use in participants in the STGBB arm demonstrated this BB surrogacy phenomenon. Including the daily cumulative dose of each BB used in the analysis may have provided more insights into the added benefit of STGBBs compared to other BBs in reducing the MAP.

## 6 Conclusions

Second- and third-generation BBs effectively reduced the MAP in hypertensives of African ancestry as well as in other racial groups. Compared with other racial groups, the risk of stroke was not increased in hypertensives of African descent who were prescribed STGBBs. However, the risk of myocardial infarction was higher in hypertensives of African descent on STGBBs. This IPD-MA suggests that the cardiovascular outcomes associated with STGBB use in managing essential hypertension may differ according to ethnicity and generation of BB therapy.

## Data Availability

Data was obtained from the Biologic Specimen and Data Repository Information Coordinating Center (BioLINCC), and requests to access the data should be directed towards the data repository. Further enquiries can be directed to the corresponding author.
