# Supplementary material for: Efficacy of beta-blockers on blood pressure control and morbidity and mortality endpoints in hypertensives of African ancestry: an individual patient data meta-analysis
Source: Front Cardiovasc Med. 2024 Jan 23;10:1280953. doi: 10.3389/fcvm.2023.1280953 (PMC10844441; doi:10.3389/fcvm.2023.1280953)
Supplement: Supplementary file 1 [file Datasheet1.docx]

**Supplemental Material**

**Efficacy of beta-blockers on blood pressure control and morbidity and mortality endpoints in hypertensives of African ancestry: An individual patient data meta-analysis (IPD-MA)**

Supplementary 1: Search Strategy

Supplementary 2 Description of dataset custodian approach for collaboration

Supplementary 3 Description of Study Characteristics, Variables, Definition Discrepancies

Supplementary 4 Risk of Bias 2 assessment

Supplementary 5 Multivariable Blood Pressure Reduction Model Assumption Checks

**Supplementary Material 1: Search Strategy**

A literature search was done in PubMed (including MEDLINE), the World Health Organization (WHO) Clinical Trials Registry (ICTRP), Cochrane Hypertension Specialised Register, Cochrane Central Register of Controlled Trials (CENTRAL), Pan African clinical Trials Registry (PACTR), ClinicalTrials.gov, Embase and database repositories including the Biologic Specimen and Data Repository Information Coordinating Center (BioLINCC), Vivli, Yale University Open Data Access Project (YODA), Mendeley Data, UK Data Service, Dryad, Harvard Dataverse, Synapse.org, Project Data Sphere, OSFHome, Centres for Disease Control and Prevention Datasets, Novo Nordisk Datasets, Data.World, ClinicalStudyDataRequest.com and The Virtual International Cardiovascular and Cognitive Trials Archive.

**Bibliographic databases** of (un)published trials were searched using automatic search strategies.

**Dataset Databases** were searched manually.

**Treatment-specific** search terms

1. Beta blocker
2. Beta-blocker
3. β blocker
4. β-blocker
5. Beta-1 blocker
6. β-1 blocker
7. β1 blocker
8. β1 antagonist
9. β-1 antagonist
10. β antagonist
11. β-antagonist
12. Adrenergic beta-antagonist (MeSH)
13. Adrenergic β-antagonist (MeSH)
14. β-adrenergic blocker
15. betaxolol
16. acebutolol
17. esmolol
18. atenolol
19. bisoprolol
20. metoprolol
21. bevantolol
22. celiprolol
23. nebivolol
24. carvedilol
25. labetalol
26. bucindelol
27. nadolol
28. penbutolol
29. pindolol
30. propranolol
31. sotalol
32. timolol
33. tertalol
34. oxprenolol

**Outcome-specific** search terms

1. Mortality
2. Cardiovascular mortality
3. Death
4. Myocardial infarction
5. MI
6. Heart failure
7. Congestive heart failure
8. Arrhythmia
9. Stroke
10. Stroke protection
11. Unstable angina
12. Hospitalisation
13. Hospitalization
14. Coronary artery disease
15. Ischaemic heart disease
16. Ischemic heart disease
17. Angina
18. Cerebrovascular accident
19. Transient ischaemic attack
20. Transient ischemic attack
21. Blood pressure
22. Hypertension
23. Arterial hypertension
24. Aortic pressure
25. End organ damage
26. Left ventricular hypertrophy
27. Proteinuria
28. Retinopathy
29. Kidney disease

**Population-specific** search terms:

1. Black
2. African
3. Black African
4. Black-African
5. African American
6. African-American
7. African descent
8. Afro-American
9. African Caribbean
10. African-Caribbean
11. Afro-Caribbean
12. Negro
13. Negroid

*See <http://dx.doi.org/10.1136/jech.2005.035964>

**Search terms will be combined as follows:**

• Primary outcome #1:

o Search terms 1-26 AND 35-39, 43, 44, 48, 49, 50, 52, 53, 54 AND 64-76

• Secondary outcome #1:

o Search terms 1-26 AND 35-39, 43, 44, 48, 49, 50, 52, 53, 54

• Secondary outcome #2:

o Search terms 1-26 AND 35-63

• Secondary outcome #3:

o Search terms 1-26 AND 35-63 AND 64-76

• Secondary outcome #4:

o Search terms 1-34 AND 35-63 AND 64-76

**Time period**: 1980-Present

**Search completed**:

- Phase 1: 20-22 July 2022
- Additional search completed for any newer publications: 18 October 2022

**Supplementary 2 Description of dataset custodian approach for collaboration**

A generic version of the email we sent to prospective collaborators.

*Dear Professor ***** *****

*I trust you are well. I’m contacting you on behalf of Prof Nqoba Tsabedze, Academic & Clinical Head - Division of Cardiology, Charlotte Maxeke Academic Hospital, University of the Witwatersrand, South Africa. As the responsible biostatistician, I’m assisting Prof Tsabedze with a meta-analysis with the aim of studying the efficacy of beta-blockers in treating hypertension and preventing cardiovascular events specifically in the patients of African descent.*

*We are contacting you with regards to the ****** dataset.*

*Some background on the purpose of the meta-analysis:*

*Contemporary clinical guidelines on the management of uncomplicated essential hypertension have downgraded the indication of beta-blocker therapy, to be used only when cardiovascular conditions (coronary artery disease (CAD), Heart failure, Arrhythmias and in young women planning on pregnancy) dominate. This has been supported by evidence suggesting that beta blockers fail to reduce the central aortic pressure and hence are associated with increased stroke events. Despite the widespread awareness of the different efficacy of hypertensive therapy in the various races, this recommendation has been broadly applied to all racial groups.*

*We are of the opinion that the exclusion of beta-blockers as a potential therapeutic option in black patients primarily comes from the lack of large RCTs investigating the efficacy of beta-blockers in treating hypertension in black patients. The majority of RCTs where evidence for beta-blocker therapy in hypertension arose were largely dominated by non-black populations. Thus, the lack of recommendation for beta-blocker therapy in black patients results from the lack of data rather than evidence against beta-blocker use in black patients.*

*We therefore hypothesise that a systematic review and a meta-analysis of pooled individual patient data of recent and old RCTs (with a focus on black patients), investigating the efficacy of new and old beta-blockers in the treatment of hypertension may reveal new insights into the role of beta-blockers in the treatment of hypertension in the black population. Furthermore, the role of new beta-blockers in the treatment of hypertension in all populations is still to be defined as there are currently no large RCTs investigating the efficacy of these new therapies.*

*Through our research, the **** group’s trial, (comparing ***** to placebo in ***** *** **** ** systolic blood pressures.) may serve as an excellent source to retrieve individual patient level data for inclusion in our systematic review and meta-analysis. The necessary data we require is not usually published as we need specific disaggregation (by race).*

*If it is possible to obtain access to the patient level data, could you point us in the right direction?*

*If you require any additional information, please let me know, so we can assist in whatever way we can. Attached is a copy of the protocol (which has already been approved by the local Ethics Committee).*

*We look forward to a favourable response from you.*

**signed by sender**

Non-response was anticipated thus updated contact information was sought, and if required, proximal faculty administrative staff was contacted by email or phone to ensure acknowledgement of our request. Eligible datasets were requested and motivated for from respective dataset repositories. Any format of IPD was accepted aiming to reduce inconvenience for researchers providing their datasets.

Where initial contact was unsuccessful, alternative dataset custodians (research institutions or commercial entities that own the respective datasets) were contacted.

The major advantage of IPD-MA was stifled by the tedious data acquisition process. Corresponding authors of large RCTs should consider delegating a data access contact that is familiar with the IPD sharing process, an alternative contact to the traditional corresponding author. Trialists should allow access to their vital resource as their limited output capabilities bottlenecks the potential of the resources they harbour. Listed corresponding authors often replied only after a third email or phone-call to their personal assistant.

**Supplementary 3 Description of Study Characteristics, Variables, Definition Discrepancies**

| **Table S3.1:** Included datasets: | | | | |
| --- | --- | --- | --- | --- |
| **Study Name** | **Acronym** | **Contacted** | **Reply** | **IPD Received** |
| Systolic Hypertension in the Elderly Program | SHEP | Yes | Yes | Yes |
| Action to Control Cardiovascular Risk in Diabetes | ACCORD | Yes | Yes | Yes |
| Systolic Blood Pressure Intervention Trial | SPRINT | Yes | Yes | Yes |
| African American Study of Kidney Disease and Hypertension Study | AASK | Yes | Yes | Yes |
| Beta-blocker Evaluation in Survival Trial | BEST | Yes | Yes | Yes |
| Beta-Blocker Heart Attack Trial | BHAT | Yes | Yes | Yes |
| Spironolactone for Heart Failure with Preserved Ejection Fraction | TOPCAT | Yes | Yes | Yes |
| The CLEVER Study - Coreg And Left Ventricular Mass Regression | CLEVER | Yes | Yes | No |
| COREG MR Versus TOPROL-XL On The Lipid Profile Of Normolipidemic Or Mildly Dyslipidemic Patients With Hypertension |  | Yes | Yes | Yes |
| A Randomized, Double-Blind, Multicenter Study Comparing the Effects of Administration of Modified Release COREG or Placebo on Blood Pressure in Essential Hypertension Patients |  | Yes | Yes | No |
| Losartan Intervention for Endpoint Reduction in Hypertension Study | LIFE | Yes | Yes | No |
| Anglo-Scandinavian Cardiac Outcomes Trial - Blood Pressure Lowering Arm | ASCOT BPLA | Yes | Yes | No |
| Controlled Onset Verapamil Investigation of Cardiovascular End Points | CONVINCE | Yes | Yes | No |
| European Lacidipine Sudy on Atherosclerosis | ELSA | Yes | Yes | No |
| International Verapamil-Trandolapril Study | INVEST | Yes | Yes | No |
| UK Prospective Diabetes Study | UKPDS | Yes | Yes | No |
| Combination Therapy of Hypertensives to prevent cardiovascular events trial group | COPE | Yes | No | No |
| Effect of carvedilol on outcome after myocardial infarction in patients with left-ventricular dysfunction: the CAPRICORN randomised tria | CAPRICORN | Yes | No | No |
| The Cardiac Insufficiency Bisoprolol Study II | CIBIS | Yes | No | No |
| Carvedilol or Metoprolol European Trial | COMET | Yes | No | No |
| Comparative longterm effects of Nebivolol Carvedilol in Hypertensive heart-failure patients |  | Yes | No | No |
| Randomized Trial to determine the effect of Nebivolol on Mortality and cardiovascular hospital admission in elderly patients with heart failure | SENIORS | Yes | No | No |
| Effect of metoprolol CR/XL in chronic heart failure: Metoprolol CR/XL Randomised Intervention Trial in Congestive Heart Failure | MERIT HF | Yes | No | No |
| The Carvedilol Prospective Randomized Cumulative Survival | COPERNICUS | Yes | Yes | No |
| Nordic Diltiazem Study | NORDIL | Yes | Yes | No |
| Captopril Prevention Project | CAPP | Yes | Yes | No |
| CARDIOvascolari del Controllo della Pressione Arteriosa SIStolica | CARDIO-SIS | 100% White population | | |
| Heart Outcomes Prevention Evaluation Study | HOPE | Yes | Yes | No |
| Swedish Trial in Old Patients with Hypertension-2 | STOP-HYPERTENSION | Yes | Yes | No |
| Dutch Transient Ischemic Attack Trial | DUTCH TIA | Yes | Yes | No |
| The COREG And Lisinopril Combination Therapy In Hypertensive Subjects (COSMOS) Trial | COSMOS | Yes | Yes | No |
| *Green: IPD obtained  * Orange: Agreed to obtain IPD but did not received | | | | |

Green: Datasets were acquired

Green FILL Dataset Acquisition abandoned due to inability/hesitancy to finalize dataset acquisition.

| **Table S3.2:** Study Characteristics of reduced RCT list | | | | | | | | | |
| --- | --- | --- | --- | --- | --- | --- | --- | --- | --- |
|  | **BEST** | **BHAT** | **SHEP** | **AASK** | **TOPCAT** | **SPRINT** | **ACCORD** | **LIFE** | **Carvedilol vs Metoprolol/ Atenolol/Placebo** |
| **Randomised** | Yes (Bucindolol vs Placebo) | Yes (Propranolol vs Placebo) | Yes | Yes (3x2 factorial design) | Spironolactone vs Placebo | Std vs Intensive BP target | Std vs Intensive BP target | Yes (Losartan vs Atenolol) | Yes  3 datasets requested and approved. |
| **Blinded** | Yes | Yes | Yes (Chlorthalidone + reserpine or Atenolol)** | Yes (Metoprolol vs Amlodipine vs Ramipril) | Not to Beta blocker use | Not to Tx | Not to Tx | Yes | Yes |
| **Primary Population** | CHF NYHA 3 (92%) or 4 (8%) & LVEF of 35% or less | 30-69 yr old 5-21 days following a myocardial infarction | Over 60 yr olds with SPB between160-219mmHg and dbp <90mmHg | 18-70yr old African American hypertensives with GFR 20-60mL/min per 1.73m2 | Over 49 years with heart failure and LVEF of 40% or less | Hypertensives with 1 CVD risk factor | DM T2 with high risk for CVD | 55-80 year old patients with hypertension and LVH | Hypertensives |
| **Mean Follow-up time** | 2 years | 2,1 years | 4,5 years | 4.1 years (Metoprolol & Ramipril) 3.8 years (amlodipine) | 3,3 Years | 3,7 Years | 3,5 Years | 4,7 years | 1 Year |
| **African Proportion** | 23,20% | 8,70% | 14% | 100% | 8,80% | 2947 | 1127 | 6% | unknown |
| **Beta-Blocker** | Bucindolol | Propranolol | Atenolol | Metoprolol | Mixed | Mixed | Mixed | Atenolol | Carvedilol / Metoprolol/Atenolol |
| **Timeline** | May 1995- July 1999 | June 1978- October 1980 | June 1984- October 1996 | February 1995- September 2000 | August 2006 -January 2012 | October 2010 – July 2016 | 2003-2009 | June 1995 - September 2001 | Variable  (Clever)Jan 2005-2008  July 2005 July 2006  Jan 2006 to December 2007 |
| Note that ACCORD only coded Carvedilol and Metoprolol while labelling every other beta-blocker as “other beta-blocker”  Carvedilol vs Metoprolol/Atenolol/Placebo Refers to four unpublished trials that were requested and approved. Only 2/4 have been received and were not included as follow uptime < 1 year  ** SHEP used atenolol as a second-line treatment. Participants extracted were only those that received a “second-line” placebo and/or reserpine | | | | | | | | | |

GREY Dataset obtained but removed in analysis

No FILL: Included in Analysis

Green FILL Dataset Acquisition abandoned due to inability/hesitancy to finalize dataset acquisition

| **Table 3.3: Variable definitions from respective datasets obtained** | | | | | | | | |
| --- | --- | --- | --- | --- | --- | --- | --- | --- |
| **Study Name** | | **Systolic Hypertension in the Elderly Program** | **Action to Control Cardiovascular Risk in Diabetes** | **Systolic Blood Pressure Intervention Trial** | **African American Study of Kidney Disease and Hypertension Study** | **Beta-blocker Evaluation in Survival Trial** | **Beta-Blocker Heart Attack Trial** | **Spironolactone for Heart Failure with Preserved Ejection Fraction** |
| **Acronym** | | **SHEP** | **ACCORD** | **SPRINT** | **AASK** | **BEST** | **BHAT** | **TOPCAT** |
| **Age** | | Age in Years at randomisation | | | | | | |
| **Race*** | | Self-identified by participant | Self-identified by participant | Self-identified by participant | Self-identified | Identified by Interviewer | Assessed by Interviewer | Assessed by Interviewer |
| **Gender** | | Self-identified by participant | Self-identified by participant | Self-identified by participant | Identified by interviewer | Identified by Interviewer | Assessed by Interviewer | Assessed by Interviewer |
| **Smoking (currently)**** | | Self-identified by participant | Self-identified by participant | Self-identified by participant | Self-identified by participant | Self-identified by participant | Self-identified by participant | Self-identified by participant |
| **History of Diabetes** | | Has a doctor told you have diabetes? | Have you had diabetes > 3 months | Have you had diabetes > 3 months | Excluded | Elicited by interview | Has a doctor told you have diabetes? | Has a doctor told you have diabetes? |
| **History of Myocardial Infarction** | | Has a doctor told you had a Heart attack? | Have you had myocardial Infarction > 3 months history | Have you had myocardial Infarction > 3 months history | Identified on ECG | Elicited by interview | Has a doctor told you had a Heart attack? | Has a doctor told you had a Heart attack? |
| **History of Stroke** | | Has a doctor told you had a stroke | Have you had stroke > 3 months history | Have you had stroke > 3 months history | Enquired at screening about previous stroke | Elicited by interview | Has a doctor told you had a stroke | Has a doctor told you had a stroke past 90 days |
| **History of Arrhythmia** | | Physical exam | ECG diagnosis | ECG diagnosis | Baseline / screening ECG | Elicited by interview | obtained from hospital records | ECG diagnosis |
| **History of Congestive Heart Failure** | | History / Physical exam | Have you had stroke > 3 months history | Have you had stroke > 3 months history | Enquired at screening about previous Congestive Heart Failure | Elicited by interview | Elicited by Interview | Diagnosed by standard criteria based on symptoms |
| **Outcomes** |  |  |  |  |  |  |  |  |
|  | **Primary Outcome: Cardiovascular mortality, Total stroke or Total Myocardial Infarction** | Three blinded physicians reviewed clinical notes, death certificates and ECGs of suspected cardiovascular events. Two neurologists reviewed the clinical notes, death certificates and autopsy findings where available to adjudicate occurrences of stroke. | On site Healthcare worker collected documents and events were adjudicated centrally by two reviewers individually | Adjudicated by a large group adjudicators listed in the SPRINT protocol | Review committee consisting of two experts adjudicated events of "a cardiovascular nature". Events are recorded as cardiovascular if both members agree on notes and special investigations done by attending doctor | ***Defined by attending doctor. | Review committee adjudicated event's likelihood as definite A (new abnormal Q-wave), Definite B (clinical symptoms + ST elevation and/or depression + serum enzyme elevation; Probable or Possible. All events adjudicated as definite, and probable were coded as a new event | Adjudicated by a clinical end-point committee at Brigham and Women’s Hospital according to prespecified criteria, members of the committee were unaware of the study-drug assignments. |
|  | **Cardiovascular Mortality** |  |  |  |  |  |  |  |
|  | **Non-fatal stroke** |  |  |  |  |  |  |  |
|  | **Non-fatal Myocardial Infarction** |  |  |  |  |  |  |  |
|  | **Total Stroke** |  |  |  |  |  |  |  |
|  | **Total Coronary Heart Disease** |  |  |  |  |  |  |  |
|  | **Total Cardiovascular disease** |  |  |  |  |  |  |  |
|  | **Baseline Systolic Blood Pressure** | Average of 3 readings (seated) | Automated average of 3 readings while seated | Automated average of 3 readings while seated | Manual Blood Pressure reading by trained professional. Three consecutive readings. The mean of the last two readings is recorded. | One reading while seated | Average of three Blood pressure readings. | Varied between manual and automatic blood pressure reading |
|  | **Baseline Dystolic Blood Pressure** |  |  |  |  |  |  |  |
|  | **Exit Systolic Blood Pressure** | Average of two readings (seated) |  |  |  |  |  |  |
|  | **Exit Diastolic Blood Pressure** |  |  |  |  |  |  |  |
| *Race as defined by each participant ** No study quantified pack years. Only "currently smoking" was congruent and therefore used*** *BEST trial did not provide a cardiovascular mortality variable. Data was extracted as follows: Myocardial infarction was defined by events/deaths that were described as "Coronary" + "Insufficiency", "attack", "embolus", "isch(a)emia", "thrombosis", "occlusion" or "Myocardial" + "infarct", "isch(a)emia". Importantly, Pulmonary embolism was included as a cardiovascular event. Stroke was defined as any event that included "cerebrovascular" or "cerebral" + "embolus", "h(a)emorrhage", "embolism", "aneurysm" "lesion", "isch(a)emia", "thromosis". Only new events were recorded. Hospital/ casualties events of ongoing / known events were not "re-recorded" | | | | | | | | |

|  | **Active Arm** | | **Placebo Arm** |
| --- | --- | --- | --- |
| **Step 1** | Chlorthalidone | 12.5mg daily | Placebo |
|  | @ 4 weeks if blood pressure not improved | 25mg daily | Placebo equivalent |
| **Step 2:** | Atenolol | 25mg daily | Placebo |
|  | @ 4 weeks Blood pressure not improved | 50mg daily | Placebo |
| *If Beta-blocker is contraindicated or Atenolol caused side effects* | Reserpine | 0.05mg daily | Placebo |
|  | Reserpine | 0.1mg daily | Placebo |

**SHEP trial description of Comparators:**

The SHEP trial looked at the use of Chlorthalidone vs placebo. We constructed the population as follows:

The dispensing of the treatment followed a twostep (Chlorthalidone and then Atenolol or Reserpine) process in at least 4 week intervals with two dose escalations within each step.

- Note that improper dosing of Atenolol was designed in this trial.

The SHEP trial groups that were compared consisted of the Atenolol (peach colour) cohort (Atenolol use for longer than 18 months) vs the cohort shaded in blue. The placebo group that remained in step one was not included as their blood pressure did not qualify for a second step and therefore the population was inherently different to the active step two arm.

**Supplementary 4 Risk of Bias 2 assessment**

| Table 4.1 Assessment of the Risk of Bias 2 | | | | | | |
| --- | --- | --- | --- | --- | --- | --- |
| BEST (2001) | 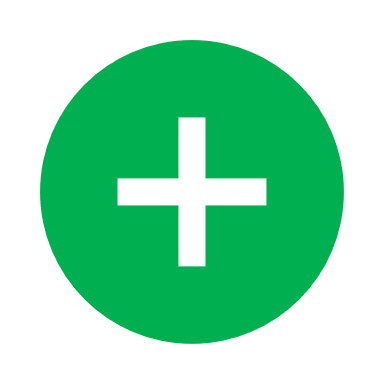 | 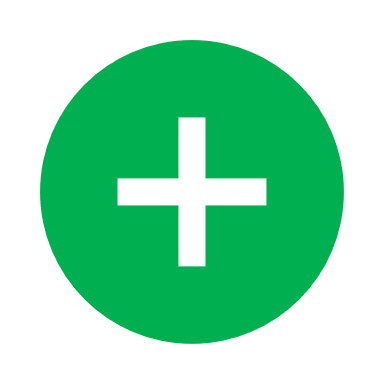 | 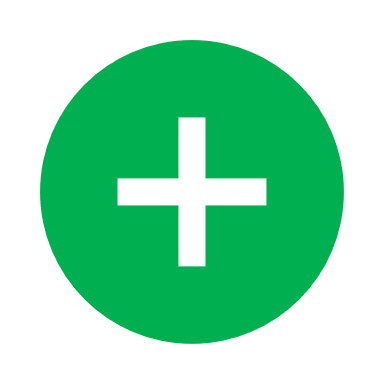 | 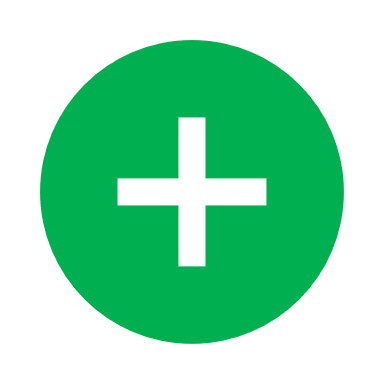 | This Domain is not applicable as a new analysis is conducted | 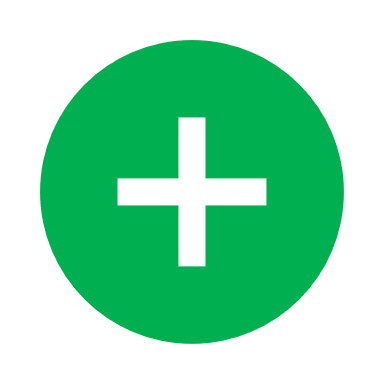 |
| BHAT (1982) | 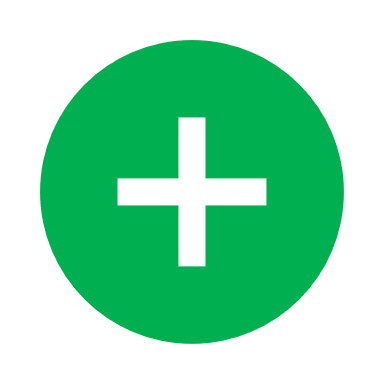 | 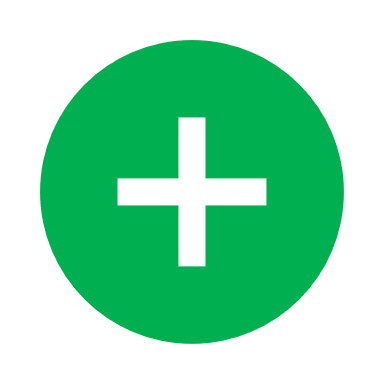 | 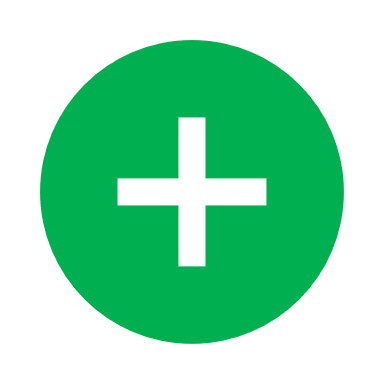 | 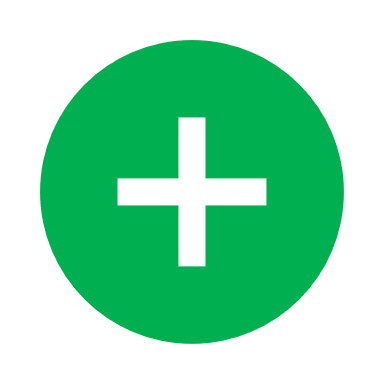 |  | 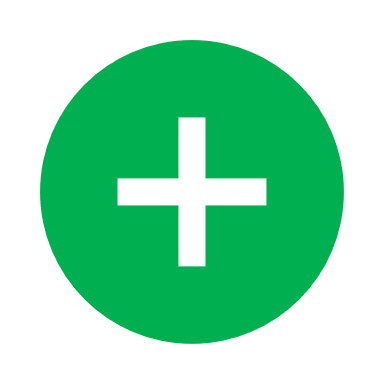 |
| SHEP (1999) | 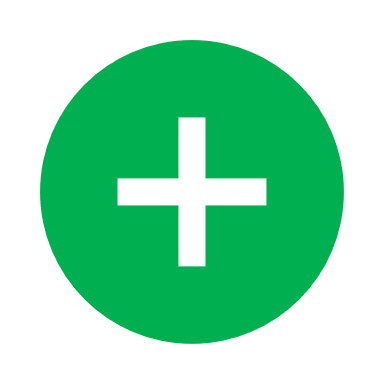 | 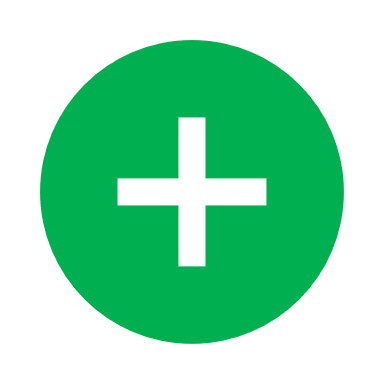 | 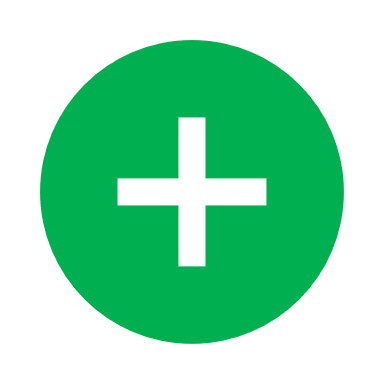 | 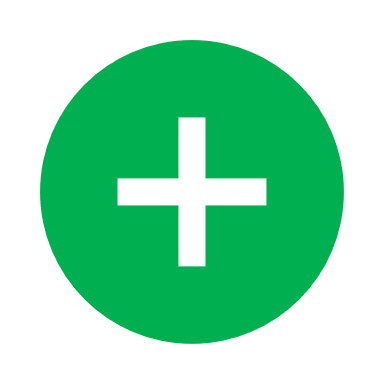 |  | 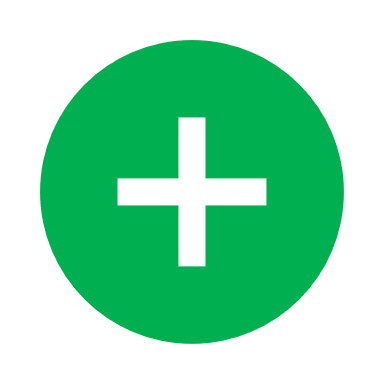 |
| AASK (2002) | 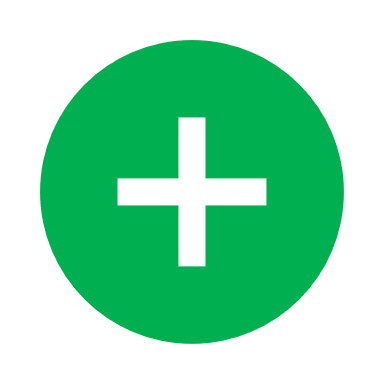 | 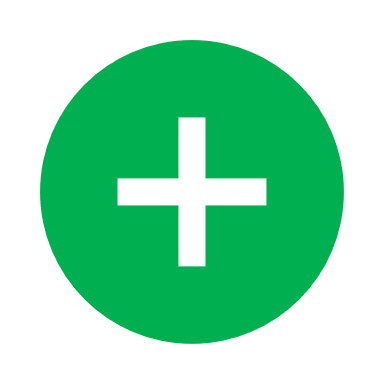 | 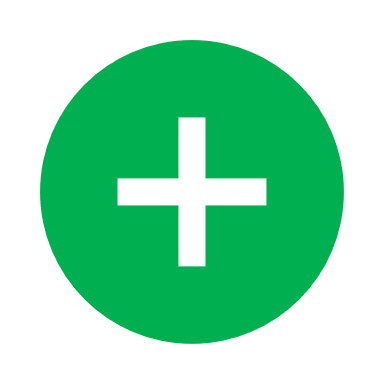 | 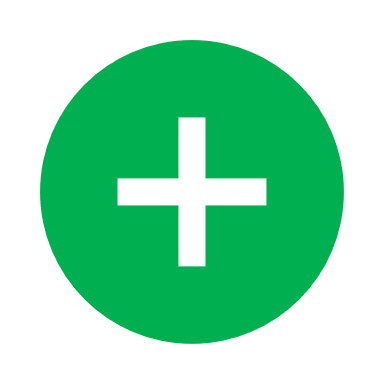 |  | 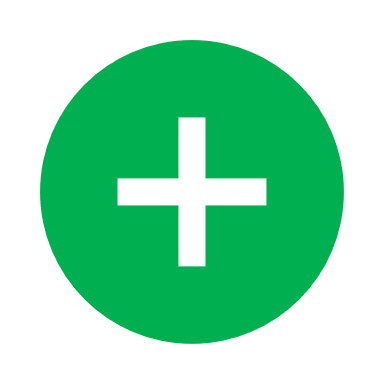 |
| TOPCAT (2014) | 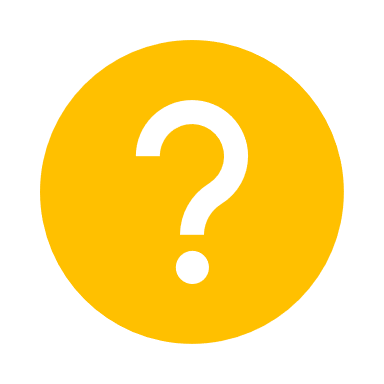 | 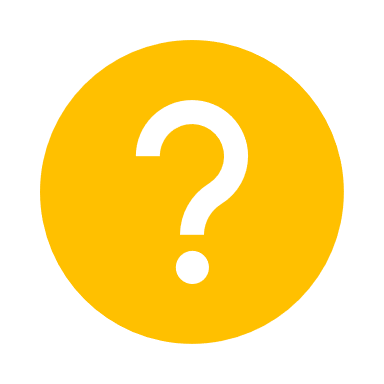 | 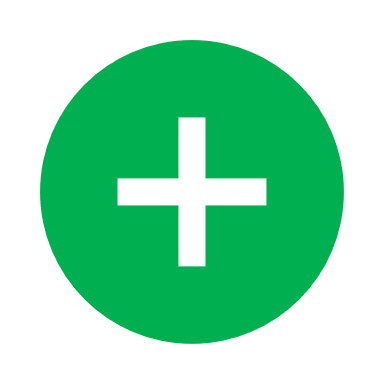 | 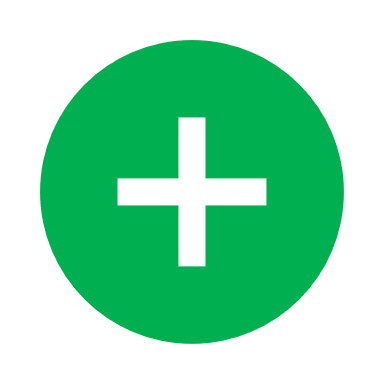 |  | 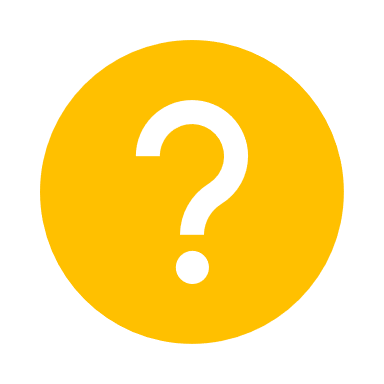 |
| ACCORD (2010) | 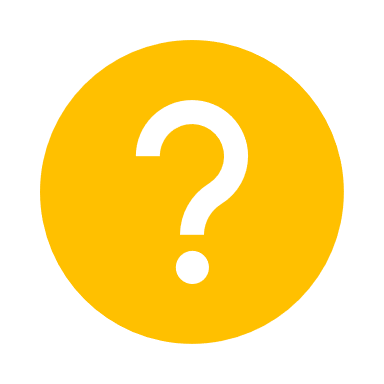 | 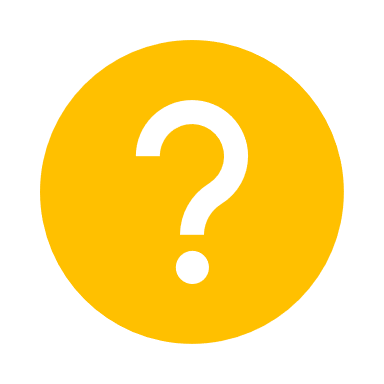 | 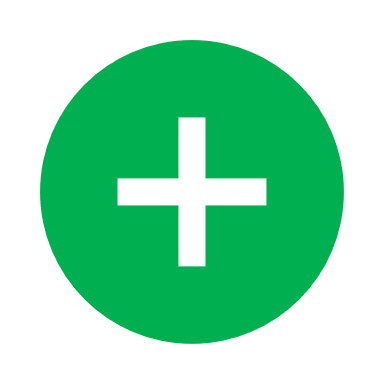 | 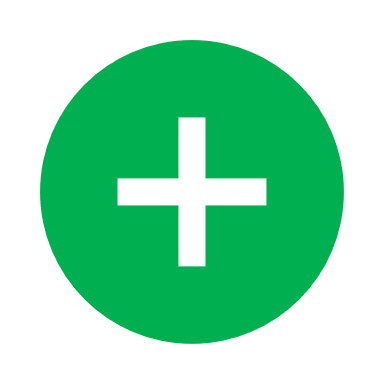 |  | 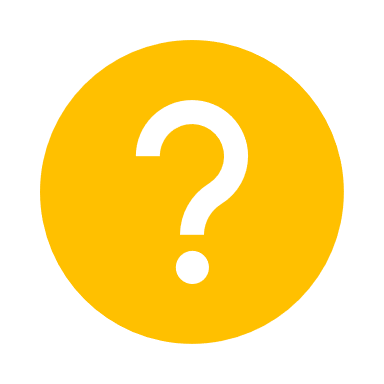 |
| SPRINT (2015) | 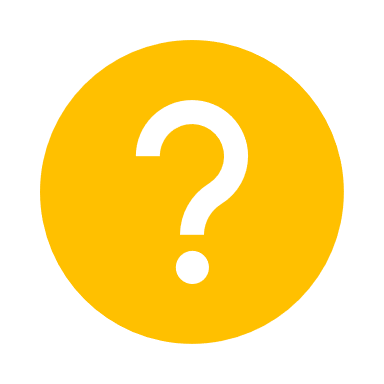 | 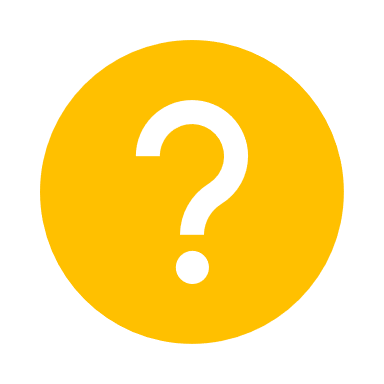 | 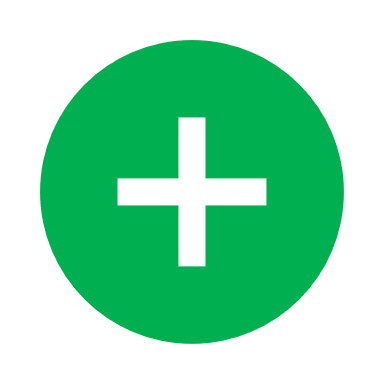 | 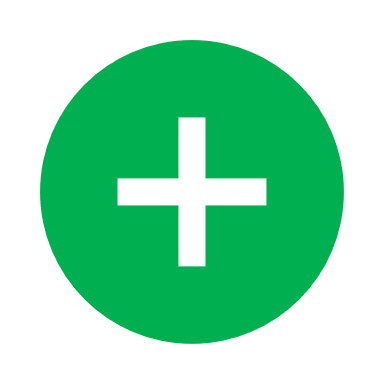 |  | 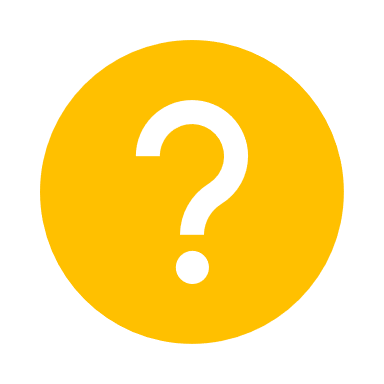 |
| The CLEVER Study - Coreg and Left Ventricular Mass Regression | 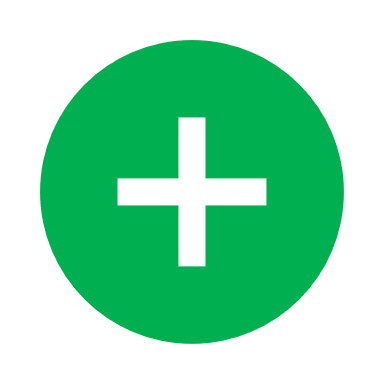 | 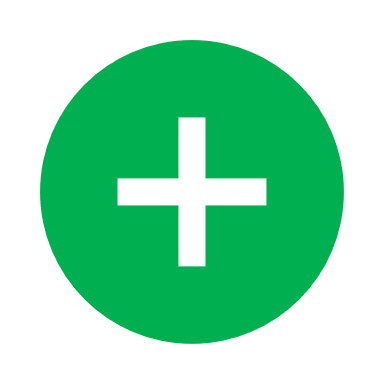 | 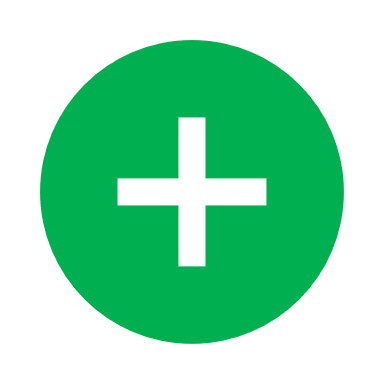 | 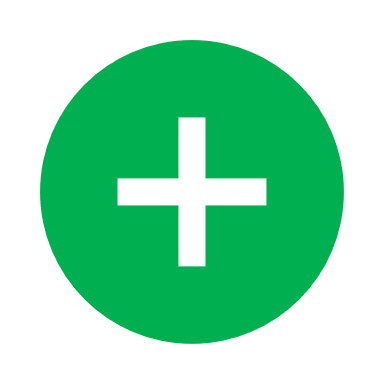 |  | 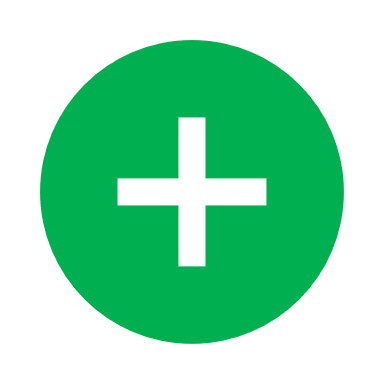 |
| COREG MR Versus TOPROL-XL on the Lipid Profile of normolipidemic or mildly dyslipidemic patients with hypertension | 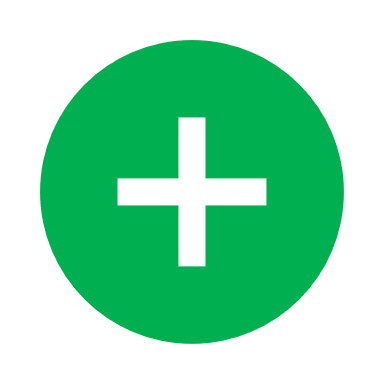 | 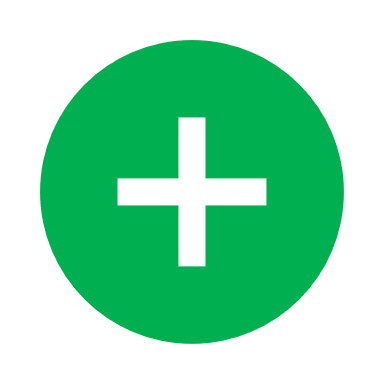 | 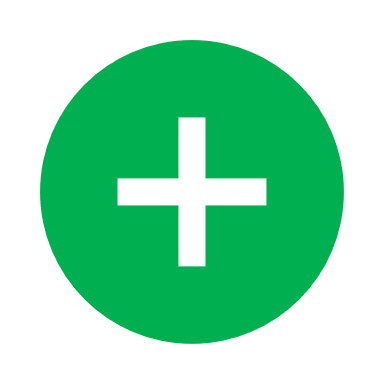 | 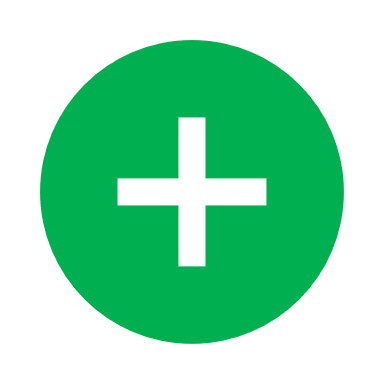 |  | 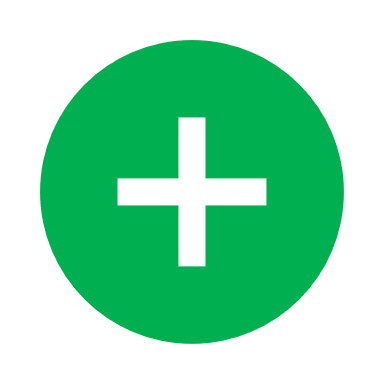 |
| A randomized, double-blind, multicentre study comparing the effects of administration of modified release COREG or placebo on blood pressure in essential hypertension patients | 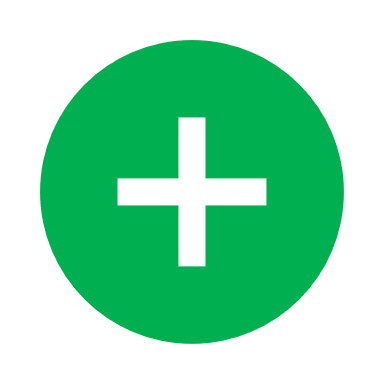 | 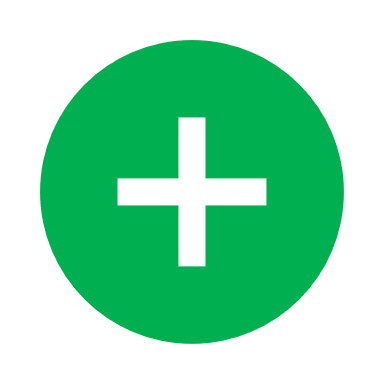 | 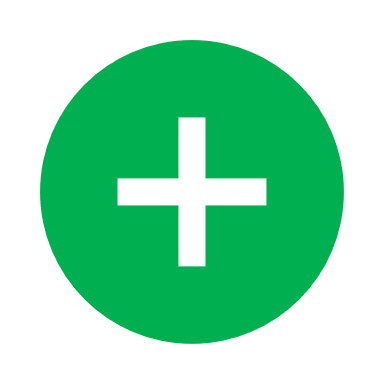 | 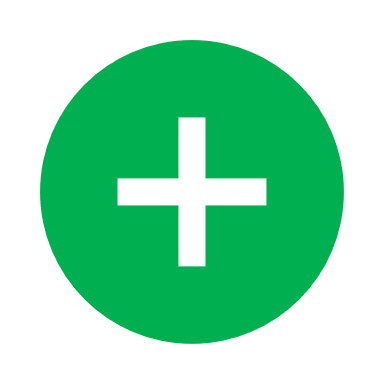 |  | 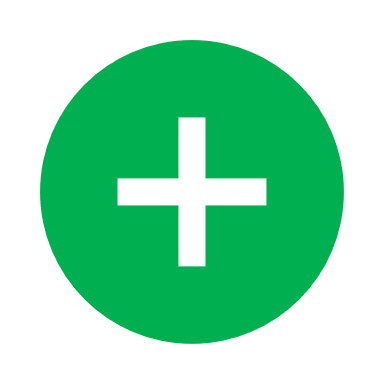 |
| LIFE (2005) | 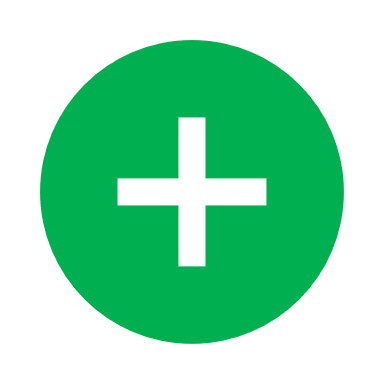 | 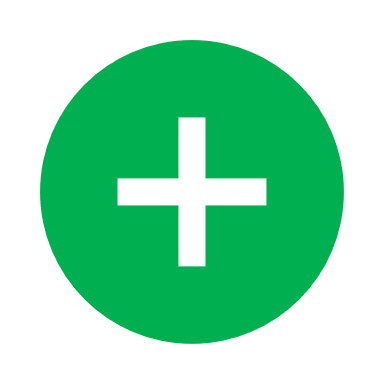 | 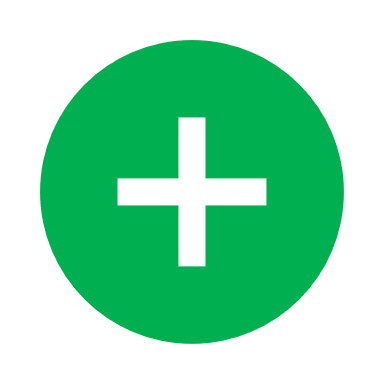 | 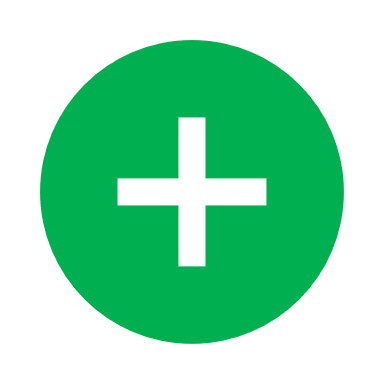 |  | 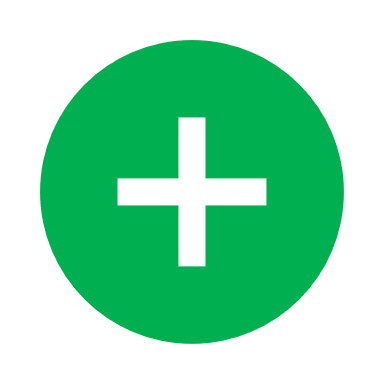 |
|  | **Domain 1: Randomisation Process** | **Domain 2: Deviations from the intended interventions (effect of assignment to intervention)** | **Domain 3: Missing outcome data** | **Domain 4: Measurement of the outcome** | **Domain 5: Selection of the reported result** | **Overall Risk of Bias assessment** |
| *Refers to three COREG MR Versus TOPROL-XL On Reduction of Microalbuminuria in Patients with Hypertension and Microalbuminuria | | | | | | |

ACCORD, SPRINT and TOPCAT were randomised focusing on treatment goal. The focus of randomisation was not focused on the use of 2^nd^ or 3^rd^ generation Beta-blockers. Although this presents a risk of bias, we do not consider this risk substantial enough to warrant an exclusion of the ACCORD, SPRINT or TOPCAT datasets.

**Supplementary 5 Multivariable Blood Pressure Reduction Model Assumption Checks**

Whole population GLMM analysis of all anti-hypertensive medications in the IPD-MA


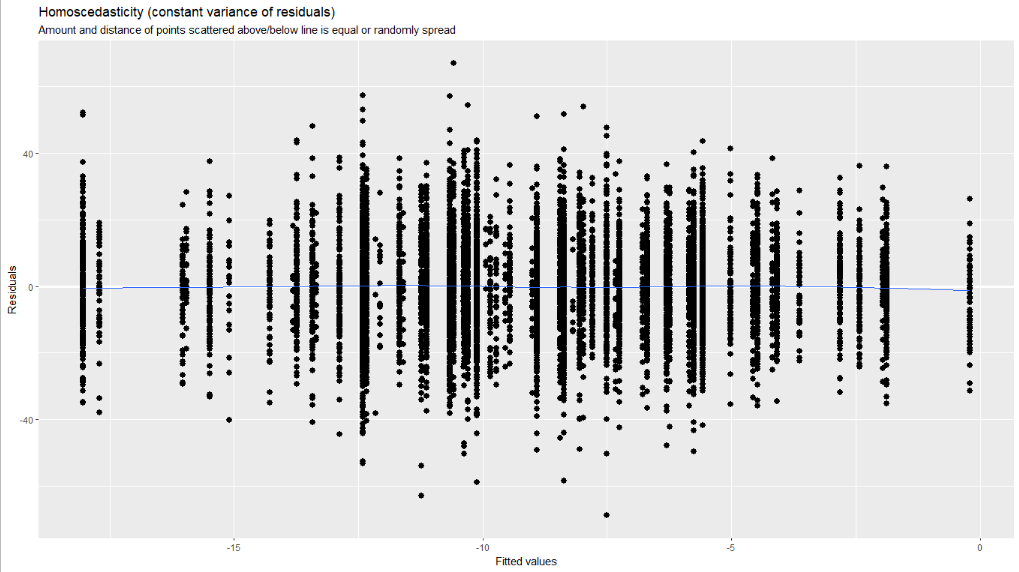


Figure 5.1: Homoscedasticity of residuals, an important assumption in any linear regression, is displayed in this figure. Since Blood pressure is measure in whole numbers and not decimals, we observe the gaps seen.


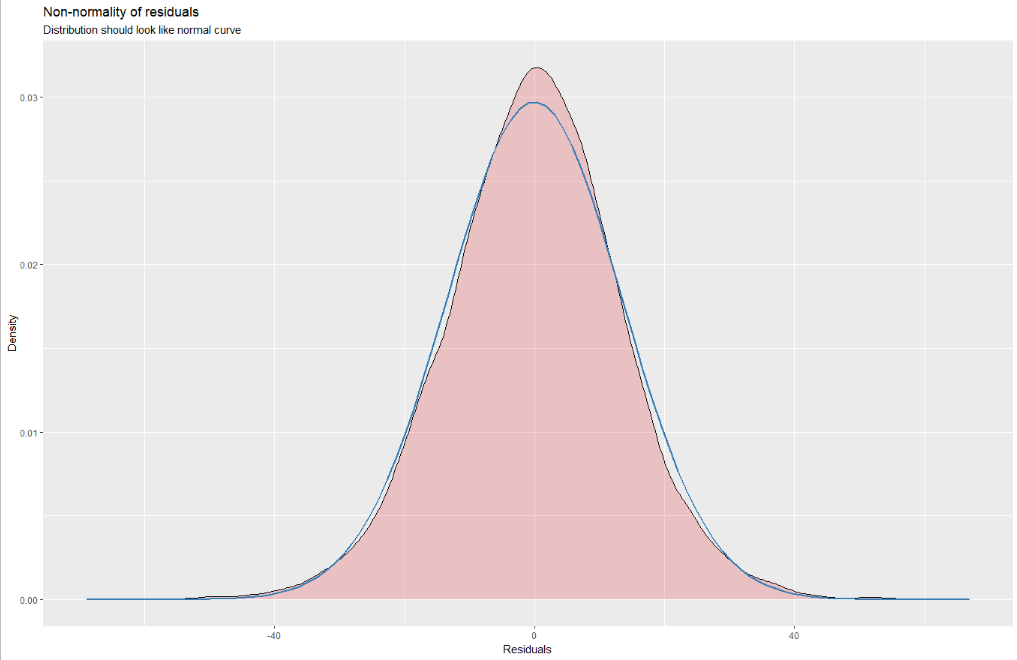


Figure 15.2: Normality of residuals is displayed in the figure above. Normality of residuals is an important assumption and is adhered to as the residuals follow a hypothetic normal distribution. The QQ-plot adheres to the theoretical normal quantile plot (not shown here).


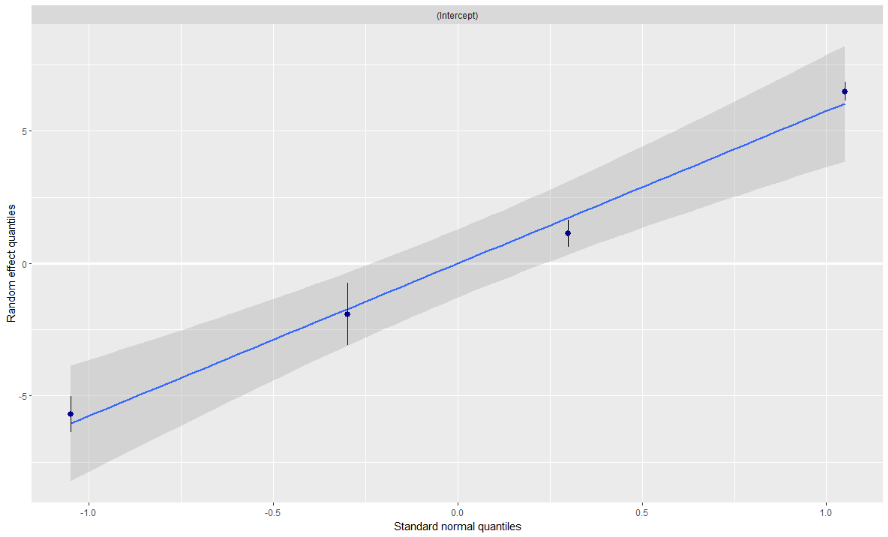


Figure 5.3: Plot of the random effect quantiles. As with the residuals, the random effects are assumed to follow a normal distribution. Albeit only four RCTs that were included, a normal distribution is demonstrated.

African population GLMM analysis of all anti-hypertensive medications in the IPD-MA


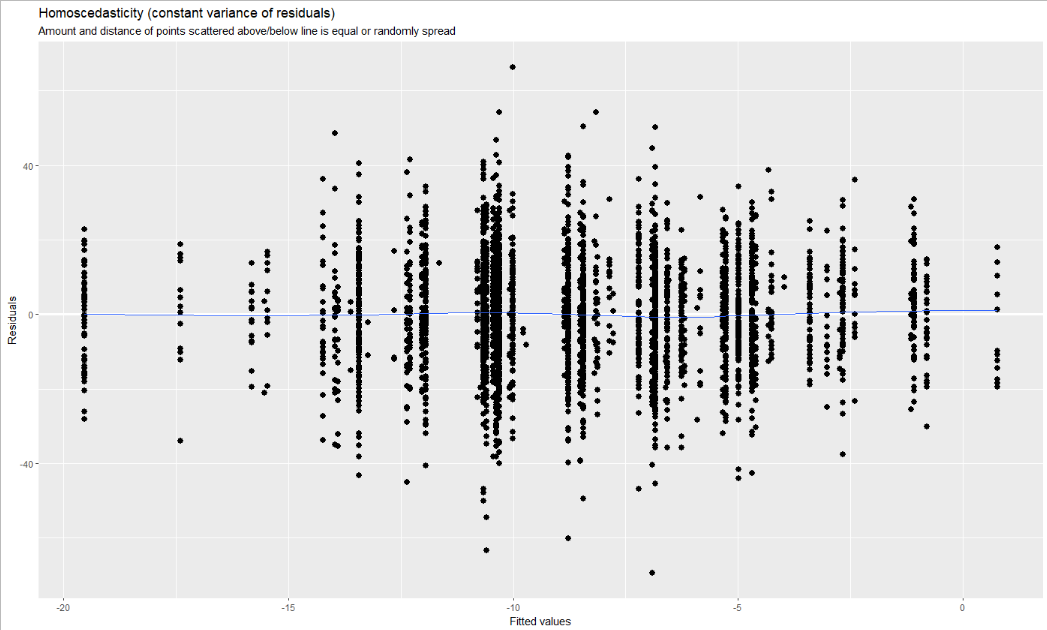


Figure 5.4: Demonstrating homoscedasticity, as with figure 5.1, but in the African population. The sample size is more than halved, explaining the paucity of residuals plotted. The Loess smoothed blue line follows the zero line closely, demonstrating homoscedasticity


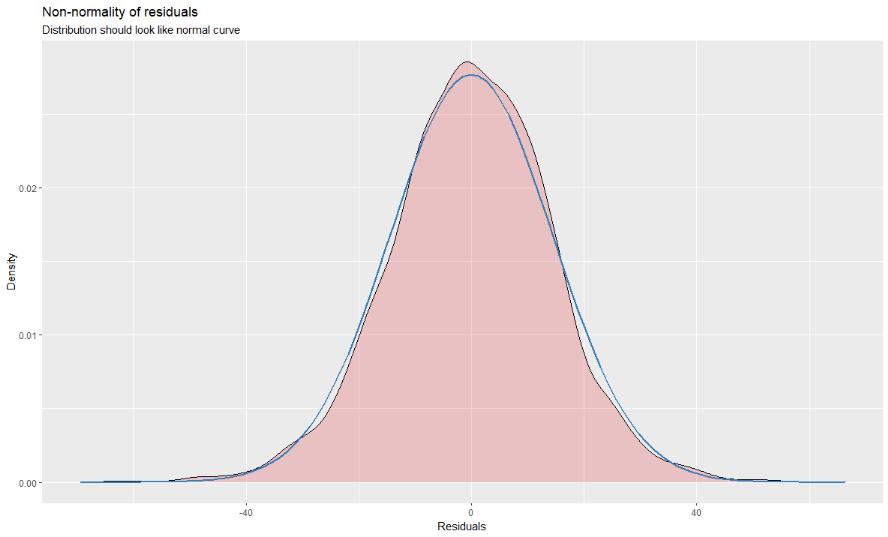


Figure 5.5: African population GLMM blood pressure lowering model demonstrating that the residuals are normally distributed. The figure demonstrates a bell curve.


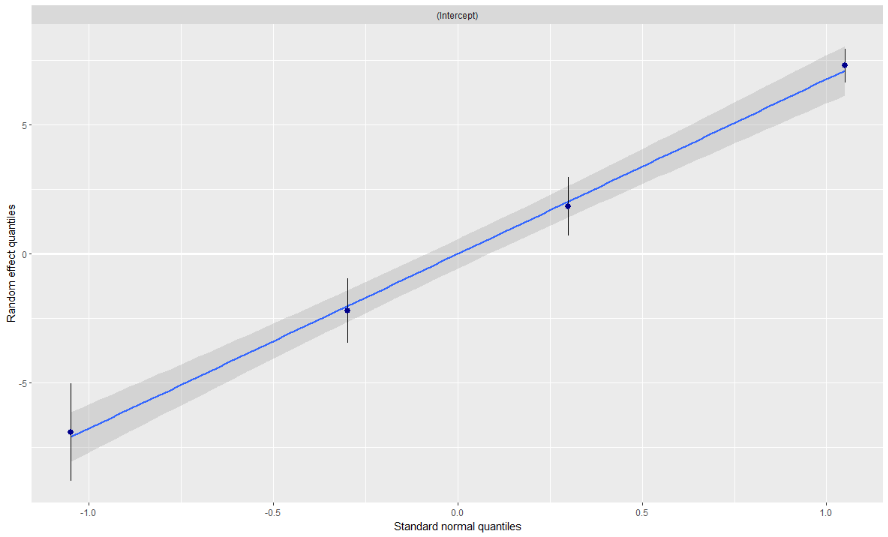


Figure 5.6: Random effects are assumed to be normally distributed. As seen by the four observations following the theoretical quantiles closely demonstrates that the random effects normality distribution has not been violated.
